# Association between blood pressure and parameters related to sleep disorders in Tabari cohort population

**DOI:** 10.1186/s40885-022-00216-3

**Published:** 2022-11-15

**Authors:** Maryam Rezapour, Mahmood Moosazadeh, Amirhossein Hessami, Mohammad Khademloo, Seyed Hamzeh Hosseini

**Affiliations:** 1 Psychiatry and Behavioral Sciences Research Center, Addiction Institute, Mazandaran University of Medical Sciences, Sari, Iran; 2 Gastrointestinal Cancer Research Center, Non-Communicable Diseases Institute, Mazandaran University of Medical Sciences, Sari, Iran; 3 Health Sciences Research Center, Addiction Institute, Mazandaran University of Medical Sciences, Sari, Iran; 4 Student Research Committee, School of Medicine, Mazandaran University of Medical Sciences, Sari, Iran; 5 Department of Community Medicine, School of Medicine, Orthopedic Research Center, Mazandaran University of Medical Sciences, Sari, Iran

**Keywords:** PERSIAN cohort, Tabari cohort, Hypertension, Sleep disorders

## Abstract

**Background:**

Insomnia and other sleep disorders can cause an increase in blood pressure, thereby resulting in premature death. Regarding this, the present study was conducted to investigate the relationship between hypertension and parameters related to sleep disorders in Tabari cohort population.

**Methods:**

In this cross-sectional study, the data from the enrollment phase of the Tabari cohort study were adopted. Tabari cohort is a part of the PERSIAN (Prospective Epidemiological Research Studies in Iran) cohort study. Data analysis was performed using descriptive and inferential statistics.

**Results:**

Out of 10,255 patients enrolled in the Tabari cohort, 2,281 patients (22.2%) had hypertension. According to the results of univariable logistic regression test, the odds ratio of high blood pressure in patients with insomnia and hypersomnia is 1.22 (95% confidence interval [CI], 1.06–1.40) and 1.22 (95% CI, 1.01–1.47) times higher than normal sleep. This odds ratio was not significant after adjusting the effect of sex, age, body mass index, waist circumference, area residence, high-density lipoprotein cholesterol, triglyceride, and total cholesterol variables with multivariable logistic regression. Frequency of routine hypnotic medication usage (14.6% vs. 5.7%, *P* < 0.001), involuntary napping (25.3% vs. 19%, *P* < 0.001), and leg restlessness during sleep (14.8% vs. 11.7%, *P* < 0.001) was higher in hypertensive individuals than in nonhypertensive cases.

**Conclusions:**

This study showed that sleep disorders prevalence are higher in hypertensive patients than nonhypertensive patients. Also, routine use of hypnotics was significantly higher medication in patients with hypertension compared to that in the nonhypertensive patients.

## Background

Sleep is an organized behavior repeated as a vital necessity based on a biological rhythm each day [1]. Sleep disorders are common among population and can have major effects on human life and health [2]. In the current highly stressed world, sleep deprivation and sleep disorders have become prevalent social problems. Accordingly, almost 30% of adults report to have some degrees of insomnia and other sleep disorders [3]. Studies from Iran has estimated that more than 40% of elderly population have sleep disorders [4]. As sleeping involve various physiological systems, disturbance in sleeping could adversely affects body including cardiovascular system [5].

Sleeping disorders potentially increases the risk of cardiovascular diseases by various physiological pathways [6]. Disorders in sleeping are considered a predictive factor for the incidence of other diseases [7] due to their considerable effect on the quality of life (e.g., absence from work or cause of traffic accidents) [8]. According to the literature, different types of physical diseases, including hypertension, can amplify this disorder [9].

Hypertension is one of the important health issues related to sleep disorders. This condition affects almost 26.4% of adults and is responsible for 13.5% of deaths. It is estimated that almost 1.5 billion of the world population will be affected by this disorder until 2025 [10]. Results of the studies by the National Foundation of Sleep in the United States in 2005 showed that along with the rise of blood pressure among the individuals in the United States, sleep hours underwent a significant decrease owing to such problems as work issues [11]. Insomnia and other sleep disorders can cause an increase in blood pressure [12, 13].

Given the important role of sleep disorders in physical and psychological health, quality of life, and its association with hypertension, this study was conducted to determine the association between hypertension and parameters related to sleep disorders in the Tabari cohort population.

## Methods

In this cross-sectional study, the data collected at the enrollment phase of the Tabari population-based cohort were used. Tabari cohort study is a part of the national cohort, named Prospective Epidemiological Research Studies in Iran (PERSIAN) [14, 15]. PERSIAN cohort is a mega cohort in Iran that has been created in different regions of Iran. In this cohort, different Iranian ethnicities are members. All Iranian ethnic groups living in areas with different climates were as criteria for entering the PERSIAN cohort study in the registration phase. Exclusion criteria included unwillingness to participate in the study, living in the designated area for less than 9 months, and physical and mental disability. The goals of the PERSIAN cohort study focus on common noncommunicable diseases such as cancers and cardiovascular disease. Also, the Tabari cohort is located in northern Iran, on the shores of the Caspian Sea. More details of the Tabari cohort are mentioned in the cohort profile [16]. The enrollment phase of the Tabari cohort included 10,255 individuals (i.e., 7,012 and 3,243 cases from urban and mountainous areas, respectively) within the age range of 35–70 years residing in urban and mountainous areas of Sari city, center of Mazandaran province, north of Iran. The instrument used in the mentioned study was a standardized questionnaire (details can be found in the methodological and cohort’s profile) [15, 16].

Anthropometric indices, including height and weight, were measured by trained individuals based on a standard protocol. The measurement of the height was accomplished using a stadiometer (SECA226, Hamburg, Germany). To this end, the individuals were asked to stand against the wall after taking off their shoes with the head straight and in line with the axis of the body and hands beside them. In addition, weight measurement was performed using an analog standing scale (SECA755).

Criteria for defining hypertension were patients with systolic blood pressure ≤ 140 mmHg or diastolic blood pressure ≤ 90 mmHg or a history of having high blood pressure or being treated with antihypertensive drugs [17]. Blood pressure was measured by two trained nurses. Barometers were calibrated. Blood pressure was measured in a sitting position after 10 min of rest, twice from the right arm and twice from the left arm (one minute between each of the two consecutive measurements). The mean of the second measurement was calculated from the right and left arm and was considered as blood pressure level. A trained supervisor at the Tabari Cohort Center monitored the blood pressure measurement process and controlled the quality of the measurement and data entry. The supervisor also used a checklist to monitor and evaluate blood pressure measurements performed by each staff member. Data registration is done on the web. According to this definition, 10,255 patients of the Tabari cohort enrollment phase results were divided into two groups of patients with and without hypertension, and all of them were included in the study by census method.

It should be noted that the definition of “short” and “long” sleep duration has been very diverse among different studies. Therefore, the criterion of sleep period < 6, 6–10, > 10 h has been determined based on the study of Cappuccio et al. [18] and the opinion of the authors of related experts.

Data analysis was carried out using IBM SPSS ver. 24 (IBM Corp., Armonk, NY, USA). The data were presented as percentage, mean, and standard deviation. The variables were compared using the chi-square test and independent-sample t-test (for comparing the mean time of daily sleep between hypertensive and non-hypertensive individuals). Logistic regression test was used to investigate the chance of insomnia and hypersomnia in hypertensive patients in the total population and based on sex, age group (35–44, 55–54, and 55–70 years), body mass index (BMI; < 25, 25–29.9, ≥ 30 kg/m^2^), place of residence (mountainous and urban areas). The significance level was defined as less than 0.05 or the absence of number 1 in the confidence interval (CI).

## Results

The present study involved the comparison of the status of sleep profiles between hypertensive and non-hypertensive patients in the whole population, as well as in terms of sex, BMI, age group, and area of residence. The results are presented separately for each variable.

### Comparison of hypertensive and non-hypertensive subjects in total population

**Fig. 1:**
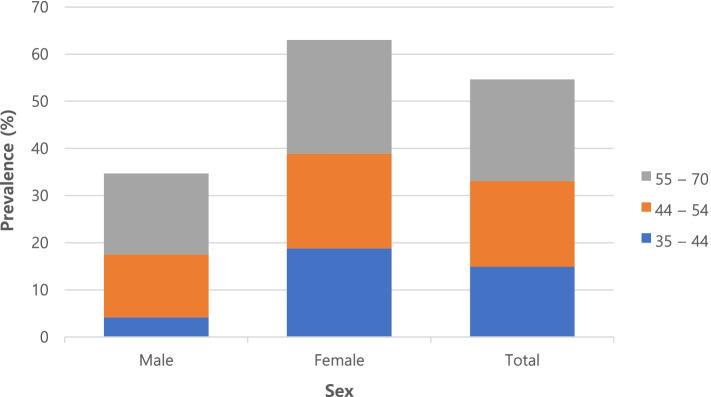
Prevalence of sleep disorders among patients with hypertension by sex and age group in Tabari cohort population

Out of 10,255 population enrolled in Tabari cohort, 2,281 patients (22.2%) had hypertension. With regard to sleep duration, 11.4%, 82.5%, and 6.1% of the patients had the sleep durations of < 6, 6–10, and > 10 h, respectively. Hypertensive cases had a higher prevalence of sleep duration of < 6 and > 10 h than the non-hypertensive cases (12.9% vs. 11% and 6.9% vs. 5.9%, respectively; *P* = 0.005). A total of 760 individuals of the Tabari cohort declared that the time interval between going to bed and sleeping was > 60 min which was higher in hypertensive patients than in non-hypertensive ones (10.4% vs. 6.5%; *P* < 0.001). Frequency of routine hypnotic medication usage (14.6% vs. 5.7%; *P* < 0.001), involuntary napping (25.3% vs. 19%; *P* < 0.001), and leg restlessness during sleep (14.8% vs. 11.7%; *P* < 0.001) was higher in hypertensive individuals than in non-hypertensive cases. In addition, the mean durations of sleep during a day were obtained as 7.53 and 7.61 h in the hypertensive and non-hypertensive subjects, respectively (*P* = 0.032) (Tables 1 and 2). The prevalence of sleep disorders among patients with hypertension by age groups of 35–44, 54–45 and 70–55 years and by total population, male patients and female patients are presented in Fig. 1. The prevalence of sleep disorders in all age groups was higher in female patients than in male patients.

**Table 1:** Comparison of sleep duration and time interval between going to bed and sleeping in hypertensive patients and non-hypertensive controls based on sex, age group, BMI, place of residence, and total population

Variable	HTN	Sleep duration (hours)						Throw sleep (hours)				
		< 6 (*n* = 1,168)	6–10 (*n* = 8,459)	> 10 (*n* = 624)	*P*-value	Mean ± SD	*P*-value	≤ 15 (*n* = 5,758)	16–30 (*n* = 2,277)	31–60 (*n* = 1,456)	> 60 (*n* = 760)	*P*-value
Sex												
M	No	84.1	81.9	75.0	0.021	7.57 ± 1.50	0.018	81.8	82.5	79.4	82.1	0.623
	Yes	15.9	18.1	25.0		7.71 ± 1.56		18.2	17.5	20.6	17.9	
F	No	70.2	75.9	74.8	0.003	7.64 ± 1.70	< 0.001	77.1	77.8	71.1	66.1	< 0.001
	Yes	29.8	24.1	25.2		7.43 ± 1.81		22.9	22.2	28.9	33.9	
Age (years)												
35–44	No	91.4	92.2	92.8	0.848	7.76 ± 1.53	0.308	92.6	91.9	90.2	92.1	0.419
	Yes	8.6	7.8	7.2		7.66 ± 1.62		7.4	8.1	9.8	7.9	
45–54	No	78.7	80.3	77.5	0.503	7.57 ± 1.58	0.848	81.8	82.0	74.6	71.1	< 0.001
	Yes	21.3	19.7	22.5		7.56 ± 1.67		18.2	18.0	25.4	28.6	
55–70	No	63.0	62.5	55.8	0.129	7.45 ± 1.77	0.585	63.3	62.7	60.6	57	0.128
	Yes	37.0	37.5	44.2		7.48 ± 1.79		36.7)	37.3	34.9	43.0	
BMI (kg/m^2^)												
< 25	No	82.6	87.8	84.5	0.033	7.57 ± 1.63	0.070	87.7	88.4	84.0	81.6	0.044
	Yes	17.4	12.2	15.5		7.39 ± 1.71		12.3	11.6	16.0	18.4	
25–29.9	No	75.5	78.9	76.0	0.161	7.63 ± 1.58	0.320	79.3	80.0	75.2	70.3	< 0.001
	Yes	24.5	21.1	24.0		7.57 ± 1.69		20.7	20.0	24.8	29.7	
≥ 30	No	68.5	70.8	68.1	0.463	7.62 ± 1.66	0.154	72.6	73.0	65.0	60.9	< 0.001
	Yes	31.5	29.2	31.9		7.53 ± 1.79		27.4	27.0	35.0	39.1	
WC (cm)												
M < 102	No	83	85.1	81.3	0.120	7.61 ± 1.57	0.486	85.1	85.1	83	81.5	0.277
F, < 88	Yes	17	14.9	18.7		7.57 ± 1.64		14.9	14.9	17	18.5	
M ≥ 102	No	67.5	71.1	69.4	0.166	7.62 ± 1.69	0.036	72.1	74	66.4	62.3	< 0.001
F ≥ 88	Yes	32.5	28.9	30.6		7.51 ± 1.79		27.9	26	33.6	37.7	
Place of residence												
Urban	No	77.8	79.1	75.2	0.106	7.75 ± 1.57	0.748	79.09	80.7	74.4	69.8	< 0.001
	Yes	22.2	20.9	24.8		7.77 ± 1.68		20.1	19.3	25.6	30.2	
Mountainous	No	71.2	76.7	73.0	0.021	7.29 ± 1.69	0.002	78.3	76.3	71.1	67.1	< 0.001
	Yes	28.8	23.3	27.0		7.07 ± 1.75		21.7	23.7	28.9	32.9	
TG (mg/dL)												
< 150	No	77.1	80.7	78.3	0.060	7.56 ± 1.62	0.065	81.4	81.4	76.2	72.7	< 0.001
	Yes	22.9	19.3	21.7		7.47 ± 1.73		18.6	18.6	23.8	27.3	
≥ 150	No	71.3	75.2	70.9	0.071	7.69 ± 1.62	0.124	76.4	77	69.2	63.7	< 0.001
	Yes	28.7	24.8	29.1		7.60 ± 1.75		23.6	23	30.8	36.3	
HDL-C (mg/dL)												
M > 40	No	77.2	79.1	75.7	0.143	7.56 ± 1.62	0.162	80.4	80.8	72.5	71.1	< 0.001
F > 50	Yes	22.8	20.9	24.3		7.49 ± 1.69		19.6	19.2	27.5	28.9	
M ≤ 40	No	69.9	76.8	73.6	0.009	7.72 ± 1.63	0.054	77.2	77.4	74.5	64.5	< 0.001
F ≤ 50	Yes	30.1	23.2	26.4		7.59 ± 1.82		22.8	22.6	25.5	35.5	
TC (mg/dL)												
< 200	No	76.2	78.2	73.8	0.078	7.61 ± 1.61	0.540	79	79.2	73.6	69.7	< 0.001
	Yes	23.8	21.8	26.2		7.58 ± 1.70		21	20.8	26.4	30.3	
≥ 200	No	72.8	78.7	76.7	0.015	7.62 ± 1.65	0.007	80	80.3	72.7	67.2	< 0.001
	Yes	27.2	21.3	23.3		7.43 ± 1.81		20	19.7	27.3	32.8	
Total population	No	74.8	25.2	74.8	0.005	7.61 ± 1.62	0.032	79.4	79.6	73.2	68.7	< 0.001
	Yes	25.2	21.6	25.2		7.53 ± 1.74		20.6	20.4	26.8	31.3	

*BMI* body mass index, *HTN* hypertension, *SD* standard deviation, *M* male, *F* female, *WC* waist circumference, *TG* triglyceride, *HDL-C* high-density lipoprotein cholesterol, *TC* total cholesterol

**Table 2:** Comparison of the routine use of sedative medications, involuntary napping, and leg restlessness during sleep between hypertensive cases and non-hypertensive controls based on sex, age group, BMI, place of residence, and total population

Variable	HTN	Has sedative routine use			Has nap involuntary at rest			Leg restlessness while sleep			
		Yes (*n* = 758)	No (*n* = 9,466)	*P*-value	Yes (*n* = 2,093)	No (*n* = 8,158)	*P*-value	Yes (*n* = 1,267)	No (*n* = 8,513)	I do not know (*n* = 471)	*P*-value
Sex											
M	No	63.8	82.6	< 0.001	78.0	82.7	0.002	78.6	82.4	76.2	0.024
	Yes	36.2	17.4		22.0	17.3		21.4	17.6	23.8	
F	No	55.5	77.1	< 0.001	68.8	76.7	< 0.001	70.6	76.0	71.6	0.002
	Yes	44.5	22.9		31.2	23.3		29.4	24.0	28.4	
Age (yr)											
35–44	No	74.8	93.0	< 0.001	89.7	92.5	0.054	89.2	92.4	93.2	0.113
	Yes	25.2	7.0		10.3	7.5		10.8	7.6	6.8	
45–54	No	62.5	81.5	< 0.001	79.3	80.2	0.623	80.9	79.9	78.8	0.853
	Yes	37.5	18.5		20.7	19.8		19.1	20.1	21.2	
55–70	No	45.9	63.9	< 0.001	61.3	62.5	0.519	57.5	63.2	59.0	0.030
	Yes	54.1	36.1		38.7	37.5		42.5	36.8	41.0	
BMI (kg/m^2^)											
< 25	No	61.5	88.9	< 0.001	85.8	87.4	0.340	83.2	88.1	79.4	0.002
	Yes	38.5	11.1		14.2	12.6		16.8	11.9	20.6	
25–29.9	No	63.2	79.5	< 0.001	71.6	80.0	< 0.001	73.8	79.3	71.6	0.001
	Yes	36.8	20.5		28.4	20.0		26.2	20.7	28.4	
≥ 30	No	49.7	72.4	< 0.001	63.8	72.1	< 0.001	66.2	71.0	70.3	0.113
	Yes	50.3	27.6		36.2	27.9		33.8	29.0	29.7	
WC (cm)											
M < 102	No	66	85.8	< 0.001	80.7	85.7	< 0.001	82.6	85.3	78.1	0.004
F < 88	Yes	34	14.2		19.3	14.3		17.4	14.7	21.9	
M ≥ 102	No	52.3	72.5	< 0.001	64.5	72.2	< 0.001	65.1	71.5	68.5	0.003
F ≥ 88	Yes	47.7	27.5		35.5	27.8		34.9	28.5	31.5	
Place of residence											
Urban	No	59.4	80.4	0.001	72.6	79.7	0.001	76.8	78.9	80.2	0.387
	Yes	40.6	19.6		27.4	20.3		23.2	21.1	19.8	
Mountainous	No	53.2	77.4	0.001	72.3	77.4	0.002	69.5	78.1	68.0	0.001
	Yes	46.8	22.6		27.7	22.6		30.5	21.9	32.0	
TG (mg/dL)											
< 150	No	58.8	81.7	< 0.001	75.1	81.4	< 0.001	77.4	80.8	75.5	0.015
	Yes	41.2	18.3		24.9	18.6		22.6	19.2	24.5	
≥ 150	No	56.2	76.1	< 0.001	68.6	75.9	< 0.001	68.2	75.7	69.6	< 0.001
	Yes	43.8	23.9		31.4	24.1		31.8	24.3	30.4	
HDL-C (mg/dL)											
M > 40	No	61.3	80.1	< 0.001	73.8	80	< 0.001	74.7	79.6	74	< 0.001
F > 50	Yes	38.7	19.9		26.2	20		25.3	20.4	26	
M ≤ 40	No	50.7	78	< 0.001	69.5	77.3	< 0.001	70.8	76.8	71.5	0.014
F ≤ 50	Yes	49.3	22		30.5	22.7		29.2	23.2	28.5	
TC (mg/dL)											
< 200	No	56.2	79.4	< 0.001	73.2	78.8	< 0.001	72.8	78.6	73.4	< 0.001
	Yes	43.8	20.6		26.8	21.2		27.2	21.4	26.6	
≥ 200	No	59.7	79.5	< 0.001	71.2	79.6	< 0.001	74.3	78.8	72.9	0.020
	Yes	40.3	20.5		28.8	20.4		25.7	21.2	27.1	
Total population	No	57.6	79.4	< 0.001	72.5	79.1	< 0.001	73.4	78.7	73.2	< 0.001
	Yes	42.4	20.6		27.5	20.9		26.6	21.3	26.8	

*BMI* body mass index, *HTN* hypertension, *M* male, *F* female, *WC* waist circumference, *TG* triglyceride, *HDL-C* high-density lipoprotein cholesterol, *TC* total cholesterol

### Comparison of hypertensive and non-hypertensive subjects based on sex

#### Male patients

Out of 4,149 male patients enrolled in the Tabari cohort, 758 patients (18.3%) had hypertension. Hypertensive male patients had a higher frequency of the sleep durations of < 6 and > 10 h, compared to the non-hypertensive ones (8.2% vs. 9.6% and 6.9% vs. 4.6%, respectively; *P* = 0.021). Routine hypnotic medication use (9.4% vs. 3.7%; *P* < 0.001), involuntary napping (24.4% vs. 19.3%; *P* = 0.002), and restless leg during sleep (12.5 vs. 10.3%; *P* = 0.024) were more frequent in hypertensive patients than in non-hypertensive (Tables 1 and 2).

#### Female patients

Out of 6,106 female patients enrolled in the Tabari cohort, 1,523 patients (24.9%) had hypertension. Frequency of sleep durations of < 6 and > 10 h was higher in female patients with hypertension than that in their non-hypertensive counterparts (15.2% vs. 11.9% and 6.9% vs. 6.8%, respectively; *P* = 0.003). The prevalence of routine usage of hypnotic medications (17.2% vs. 7.1%; *P* < 0.001), involuntary napping (25.7% vs. 18.8%; *P* < 0.001), and leg restlessness during sleep (15.9% vs. 12.7%; *P* = 0.002) was also higher in hypertensive females as compared to those in the non-hypertensive females (Tables 1 and 2).

### Comparison of hypertensive and non-hypertensive subjects based on age group

A total of 3,331 patients enrolled in the Tabari cohort were within the age group of 35–44 years. In this population, 262 patients (7.9%) suffered from hypertension. In this group, hypertensive patients had a lower frequency of sleep durations of < 6, 6–20, and > 10 h than the non-hypertensive individuals (9.2% vs. 8.3% and 5.7% vs. 6.3%, respectively; *P* = 0.848). Prevalence of the routine usage of hypnotic medications (15.6% vs. 4%; *P* < 0.001), involuntary napping (15.6% vs. 11.6%; *P* = 0.054), and restless leg during the sleep (13.4% vs. 9.4%; *P* = 0.113) was higher in hypertensives than in non-hypertensives (Tables 1 and 2).

With regard to the age group of 45–54 years, 3,379 patients were within this age group out of whom 676 patients (20%) had hypertension. Prevalence of sleep durations of < 6 and > 10 h was lower in the hypertensive cases than in the non-hypertensive ones (11.8% vs. 10.9% and 6.4% vs. 5.5%, respectively; *P* = 0.503). Routine use of hypnotic medications (14.9% vs. 6.2%; *P* < 0.001) and involuntary napping (20.6% vs. 19.7%; *P* = 0.623) were more prevalent in hypertensives as compared to those in non-hypertensives (Tables 1 and 2).

A total of 3,545 subjects enrolled in the Tabari cohort were within the age group of 55–70 years. In this population, 1,343 patients (37.9%) suffered from hypertension. In this age group, the hypertensive patients had a higher frequency of sleep durations of < 6 and > 10 h in comparison to the non-hypertensives (14.1% vs. 14.7% and 7.4% vs. 5.7%, respectively; *P* = 0.129). The prevalence of the routine use of hypnotic medications (14.2% vs. 7.4%; *P* < 0.001) and involuntary napping (29.5% vs. 28.5%; *P* = 0.519) was higher in the hypertension group than in the non-hypertensive group (Tables 1 and 2).

### Comparison of hypertensive and non-hypertensive subjects based on BMI and waist circumference

Based on the data, 2,473 patients had a BMI of < 25 kg/m^2^. Out of this group, 321 patients (13%) suffered from hypertension. Prevalence of sleep durations of < 6 and > 10 was higher in the hypertensives in this group than in their non-hypertensive counterparts (15.6% vs. 11% and 6.2% vs. 5.1%, respectively; *P* = 0.033). Furthermore, the routine use of hypnotic medications (20.2% vs. 4.8%; *P* < 0.001) and involuntary napping (23.1% vs. 20.7%; *P* = 0.340) were more frequent in the hypertensive group (Tables 1 and 2).

With regard to the BMI of 25–29.9 kg/m^2^, a total of 4,343 individuals enrolled in the Tabari cohort were identified to be located in this group, 941 (21.7%) of whom had hypertension. Hypertensive individuals with a BMI of 25–29.9 kg/m^2^ had higher rates of sleep durations of < 6 and > 10 in comparison with their non-hypertensive counterparts (12.4% vs. 10.6% and 6.7% vs. 5.9%, respectively; *P* = 0.161). Routine use of hypnotic medications (12.1% vs. 5.8%; *P* < 0.001) and involuntary napping (26% vs. 18.2%; *P* < 0.001) were more prevalent in the hypertensive group than in the non-hypertensive control (Tables 1 and 2).

In addition, 3,439 patients admitted to the Tabari cohort had a BMI of ≥ 30 kg/m^2^, 1,019 (29.6%) of whom had hypertension. Frequency of the sleep durations of < 6 and > 10 h in the hypertensive subjects with a BMI of ≥ 30 kg/m^2^ was higher in comparison to that in their non-hypertensive counterparts (12.5% ​​vs. 11.4% and 7.3% vs. 6.5%, respectively; *P* = 0.463). The prevalence of the routine use of hypnotic drugs (15.1% vs. 6.3%; *P* < 0.001) and involuntary napping (25.2% vs. 18.7%; *P* < 0.001) was higher in the hypertensive patients than in the non-hypertensives (Tables 1 and 2). Also, the prevalence of insomnia, hypersomnia, throw sleep, sedative routine use, nap involuntary at rest, and leg restlessness while sleep among hypertensive and non-hypertensive patients in terms of waist circumference (WC) is presented in Tables 1 and 2.

### Comparison of hypertensive and non-hypertensive subjects based on place of residence

#### Urban population

A total of 7,012 patients enrolled in the Tabari cohort were from urban areas. Out of this population, 1,493 (21.3%) had hypertension. Frequency of sleep durations of < 6 and > 10 in the hypertensive population living in urban areas was higher, compared to those in their non-hypertensive counterparts (9.4% vs. 9% and 8.4% vs. 6.9%, respectively; *P* = 0.106). Routine use of hypnotic medications (15% vs. 5.9%; *P* < 0.001) and involuntary napping (18.8% vs. 13.5%; *P* < 0.001) had a higher frequency in hypertensives (Tables 1 and 2).

#### Mountain population

A total of 3,243 patients admitted to the Tabari cohort resided in the mountainous areas, 788 (24.3%) of whom suffered from hypertension. In this group, the frequency of sleep durations of < 6 and > 10 was higher in the hypertensive patients than in the non-hypertensive subjects (19.4% vs. 15.4% and 3.9% vs. 3.4%, respectively; *P* = 0.021). The frequency of the routine use of hypnotic drugs (13.8% vs. 5.1%; *P* < 0.001) and involuntary napping (37.6% vs. 31.5%; *P* = 0.002) was higher in non-hypertensive patients (Tables 1 and 2).

### Lipid profile

The prevalence of sleep duration less than 6 h and more than 6 h, sedative routine use, nap involuntary at rest, and leg restlessness while sleep in patients with high triglyceride (TG) was 28.7%, 29.1%, 43.8%, 31.4% and 31.8% respectively. More details on the prevalence of factors related to sleep disturbance, hypnotic drugs, and restless legs syndrome in terms of high-density lipoprotein cholesterol (HDL-C) and total cholesterol (TC) are presented in Tables 1 and 2.

### Univariable and multivariable logistic regression

According to the results of univariable logistic regression test, the odds ratio of high blood pressure in patients with insomnia and hypersomnia is 1.22 (95% CI, 1.06–1.40) and 1.22 (95% CI, 1.01–1.47) times higher than normal sleep respectively and in patients with sleep disorders 1.22 (95% CI, 1.08–1.37) times more than patients without sleep disorders. The results of multivariable logistic regression, which shows the odds ratio of hypertension in patients with sleep disorders, after adjusting for the effect of sex, age, BMI, WC, area residence, HDL-C, TG, and TC variables, are presented in Table 3.

**Table 3:** Relationship between sleep disorders and blood pressure using univariable and multivariable logistic regression

Variable	Univariable logistic regression			Multivariable logistic regression^a)^		
	OR	95% CI	P-value	OR	95% CI	*P*-value
Sleep duration						
Normal (6–10 h)	Ref	Ref	Ref	Ref	Ref	Ref
Insomnia (< 6 h)	1.22	1.06–1.40	0.006	0.99	0.85–1.16	0.938
Hypersomnia (> 10 h)	1.22	1.01–1.47	0.040	1.13	0.92–1.38	0.241
Sleep disorders						
No	Ref	Ref	Ref	Ref	Ref	Ref
Yes	1.22	1.08–1.37	0.001	1.04	0.91–1.18	0.561

*OR* odds ratios, *CI* confidence interval, *Ref*. reference

^a)^Adjusted by sex, age, body mass index, waist circumference, area residence, high-density lipoprotein cholesterol, triglyceride, and total cholesterol

## Discussion

The present study involved the investigation of the association between sleep parameters and hypertension in Tabari cohort study. The odds of insomnia were 22% higher in the patients with hypertension than in the non-hypertensive cases. Furthermore, regarding hypersomnia in the total population, the odds ratio of this variable was significantly 22% higher in hypertensive patients as compared to that in the non-hypertensives. Routine use of hypnotic medications was significantly higher in hypertensive patients in all categorizations, namely sex, all age groups, place of residence, and BMI, in comparison to those in their non-hypertensive counterparts.

The pathophysiological mechanism behind sleep disorders and blood pressure has not been fully understood however there are possible pathways which might explain it. In normal sleeping there is a 10% to 20% decrease in blood pressure, which is being referred as “nocturnal dipping.” Sleep disorder may cause disturbance in nocturnal dipping which predispose individuals to cardiovascular diseases including hypertension [19]. Activated sympathetic nervous system and hypothalamic-pituitary-axis during sleep disorders could also contribute to hypertension in these patients [20]. Increase in inflammatory cytokines was associated with sleep loss, could also damage cardiovascular endothelial and predispose patients to cardiovascular disorders [21, 22].

The association between hypertension and sleep disorders has been investigated in multiple studies. In a cohort study by Zou et al. [23] investigating the association between hypertension and reduced sleep time, showed that decreased total sleep time was independently and significantly associated with hypertension. Yadav et al. [24] also investigated 1,215 individuals aged 40 to 70 years in South Korea over a follow-up period of 2.5 years. After eliminating confounding factors, their results showed that a sleep duration of > 6 h was significantly and independently associated with hypertension. However, they observed no association between hypertension and prolonged sleep time (i.e., > 8 h), which is inconsistent with our results.

In a study carried out by Sun et al. [25] on 20,505 individuals aged 35 to 64 years in China, a sleep duration of < 6 h during a day in female patients with 35 to 44 years of age increased the risk of hypertension after eliminating the confounding factors. In the present study, the odds of insomnia and hypersomnia in the hypertensive patients aged 35 to 44 years were higher, compared to those in the normal individuals. In another study conducted by Lu et al. [26] in China on male population, sleep quality and decreased sleep duration were reported to be associated with hypertension.

Shivashankar et al. [13] examined a group of patients aged > 20 years at a prevention cardiac disease center in India. In the mentioned study, the hypertension prevalence was obtained at 30%. Their results also revealed that increased blood pressure was associated with insomnia and snoring. However, hypertension was not significantly associated with the duration of sleep and daytime sleepiness. In the current study, although the prevalence of hypertension was 22%, daytime napping was higher in individuals with hypertension than in those without hypertension.

In a study performed on middle-aged females in the United States, Matthews et al. [12] showed that the duration and quality of sleep had no associations with hypertension among the investigated population. On the other hand, the examination of brain waves showed that women with increased beta waves during sleep had a higher risk of hypertension. In addition, those with a decreased delta wave during sleep were at a higher risk of diastolic hypertension. Results of the mentioned study emphasized that females who had lower than normal delta waves in Non-rapid Eye Movement (NREM) sleep were at a higher risk of developing hypertension. In the present research, increased blood pressure was associated with sleep disorders in female population; however, unlike the results obtained by Matthews et al. [12], the prevalence of hypertension was higher in population with high or low sleep duration.

Kielbasa et al. [27] showed that aging heightens the risk of sleep disorders in non-hypertensive society; accordingly, this point could be confirmed in our study. In another study performed by Javaheri et al. [28] on adolescents in Ohio, USA, low quality of sleep in young healthy adolescents was associated with prehypertensive stage. However, this factor showed no association with other factors, including socioeconomic status, obesity, and other comorbidities. This is indicative of the role of sleep quality at earlier ages.

Results of a study conducted by Jackowska et al. [29] on individuals aged > 50 years in England showed that sleep pattern disorders could cause cardiovascular problems by increasing blood pressure. In our study, with respect to the higher prevalence of hypertensive disorder in the elderly, this point should be noted that in case of treating sleep pattern disorder at an appropriate time, cardiovascular accidents can be prevented. A study conducted in Iran by Gaffari et al. [11] also showed a significant association between daytime sleepiness and such factors as age, BMI, and medication regimen.

Furthermore, in a meta-analysis carried out by Shen et al. [30] on restless leg syndrome and hypertension, the prevalence of restless leg syndrome was reported to be higher in hypertensive patients than in normal population. In the same vein, the results of our study showed that leg restlessness was more prevalent in hypertensive population. The investigation of the association between sleep duration and hypertension and the role of sleep correction in hypertension prevention is a complicated measure and requires further investigations and meta-analysis studies, especially with the respect to the discrepancies between the results reported in the studies performed all over the world.

In Isfahan Healthy Heart cohort study, the frequency of sleep duration less than 6 h, 6 h, 7–8 h, and more than 8 h in patients with high WC was 46.2%, 39.4%, 35.7%, and 34.3%, respectively. These differences were statistically significant (*P* < 0.01). The frequency of sleep less than 6 h, 6 h, 7–8 h, and more than 8 h in patients with low HDL was 43.3%, 44.2%, 45.6%, and 48%, respectively. These differences were not statistically significant (*P* > 0.05) [31]. It should be noted that these results were in line with the present study.

Lack of enough information regarding the real causal inference between sleep disorders and blood pressure is one of the limitations of the present study. Because in the present study, the data of the cohort study enrollment phase have been used in a cross-sectional method. Due to this limitation, it was not possible to judge the temporal priority of sleep disorders and blood pressure incidence. It should be noted that in the upcoming years, based on the results of the follow-up phase of the Tabari cohort, the temporal precedence of sleep disorders and blood pressure can be judged and the possible causal relationship between these two variables can be assessed.

One of the strengths of the present study is that the enrollment phase data of a large sample cohort (Tabari cohort) was used, which has a high validity. Also, the results of the present study can be suitable platform for research on sleep disorders and noncommunicable disease based on follow-up phase data of Tabari cohort study.

## Conclusions

Prevalence of routine hypnotic medication usage was also significantly higher in hypertensive patients in all categorizations, namely sex, BMI, place of residence, and total population, compared to that in the nonhypertensive patients. As the results of the present study indicated, hypertensive patients had a significantly higher level of sleep disorders in comparison to the nonhypertensive subjects.

## Data Availability

The datasets used and/or analyzed during the current study are available from the corresponding author on reasonable request.

## Abbreviations

BMI: Body mass index
HDL-C: High-density lipoprotein cholesterol
PERSIAN: Prospective Epidemiological Research Studies in Iran
TC: Total cholesterol
TG: Triglyceride
WC: Waist circumference
